# Author Correction: Feature selection and dimension reduction for single-cell RNA-Seq based on a multinomial model

**DOI:** 10.1186/s13059-020-02109-w

**Published:** 2020-07-22

**Authors:** F. William Townes, Stephanie C. Hicks, Martin J. Aryee, Rafael A. Irizarry

**Affiliations:** 1 Department of Biostatistics, Harvard University, Cambridge, MA USA; 2 Present Address: Department of Computer Science, Princeton University, Princeton, NJ USA; 3 Department of Biostatistics, Johns Hopkins University, Baltimore, MD USA; 4 Molecular Pathology Unit, Massachusetts General Hospital, Charlestown, MA USA; 5 Center for Cancer Research, Massachusetts General Hospital, Charlestown, MA USA; 6 Department of Pathology, Harvard Medical School, Boston, MA USA; 7 Department of Data Sciences, Dana-Farber Cancer Institute, Boston, MA USA

**Correction to: Genome Biol 20, 295 (2019)**

**https://doi.org/10.1186/s13059-019-1861-6**

Following publication of the original paper [1], an error was identified. In the original version of this manuscript, we referred to the Zheng 10x datasets as FACS-purified. However, based on Supplemental Table 8 of the Zheng et al. (2017) publication, the cells were actually purified by cell-type specific isolation kits that did not involve sorting by fluorescence. The authors thank Miriam Shiffman for identifying this error.
