# Supplementary material for: Feature selection and dimension reduction for single-cell RNA-Seq based on a multinomial model
Source: Genome Biol. 2019 Dec 23;20:295. doi: 10.1186/s13059-019-1861-6 (PMC6927135; doi:10.1186/s13059-019-1861-6)
Supplement: Supplementary file 1 — Additional file 1 Contains supplementary figures S1–S11 and tables S1–S4. [file 13059_2019_1861_MOESM1_ESM.pdf]

Supplemental Figures and Tables

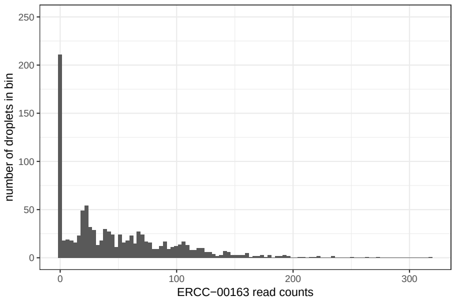

(a) Read counts- technical replicates

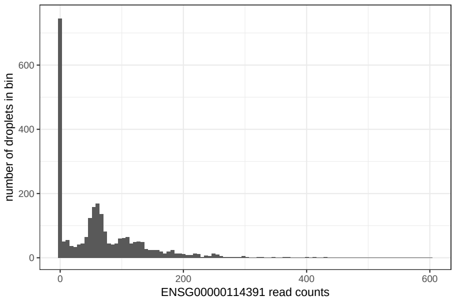

(b) Read counts- biological replicates

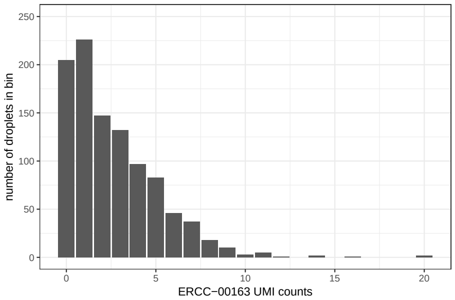

(c) UMI counts- technical replicates

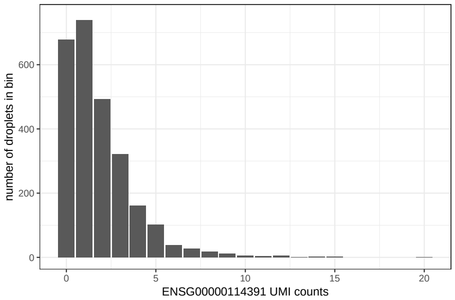

(d) UMI counts- biological replicates

Figure S1: Comparing read counts and UMI counts sampling distribution from technical and monocytes biological replicates negative control datasets. a) Read count distribution for spike-in ERCC-00163 across technical replicates. b) Read count distribution for gene ENSG00000114391 across biological replicates (purified monocytes). c) as a) but without PCR duplicates. d) as b) but without PCR duplicates.

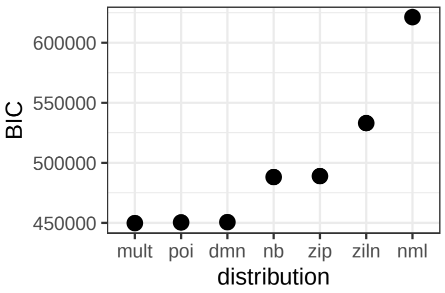

(a) Tung UMI counts

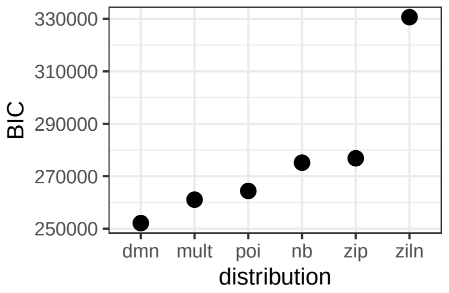

(b) Zheng monocytes UMI counts

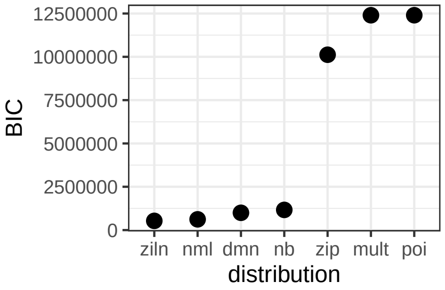

(c) Tung read counts

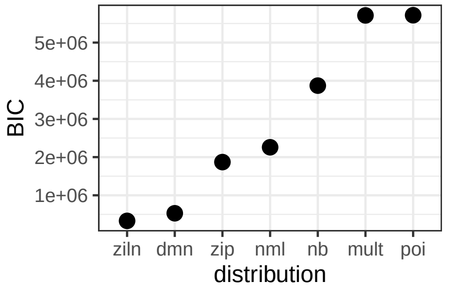

(d) Zheng monocytes read counts

Figure S2: Multinomial models provide best fit to UMI counts from biological replicates negative controls. a) Bayesian information criterion (BIC, lower=better) from maximum likelihood fits of different null distributions to the UMI counts from the Tung dataset. b) as a) but with the Zheng monocytes dataset. c) as a) but without removal of PCR duplicates. d) as b) but without removal of PCR duplicates. dmn: Dirichlet-multinomial, ziln: zero-inflated lognormal, mult: multinomial, poi: Poisson, nb: negative binomial, nml: normal. zip: zero-inflated Poisson.

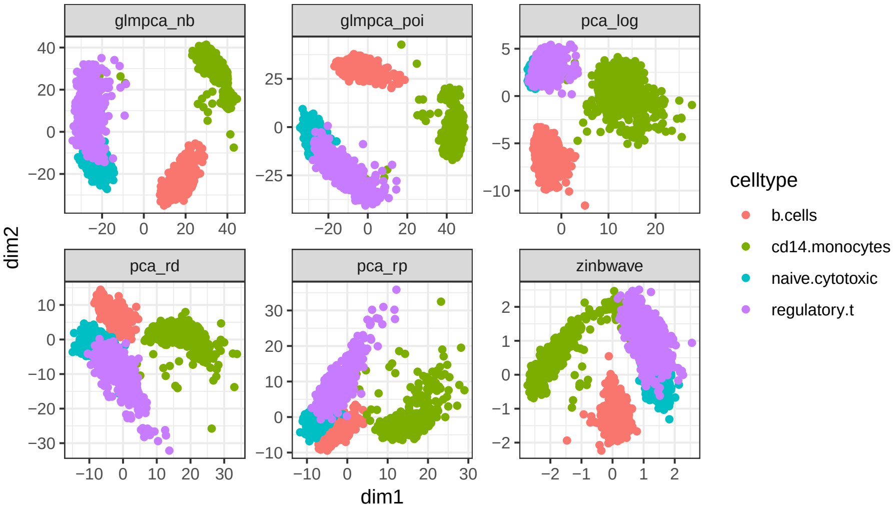

(a) Zheng 4eq

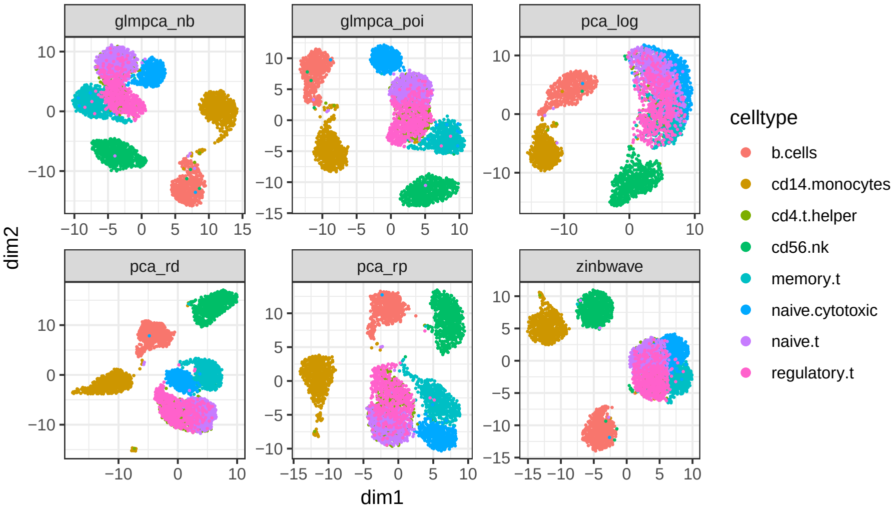

(b) Zheng 8eq

Figure S3: GLM-PCA effectively visualizes biological differences in low-dimensional space for ground truth datasets. a) Direct reduction to two dimensions for the Zheng 4eq dataset. Feature selection was 2,000 highly variable genes for pca_log and zinbwave, and 2,000 high deviance genes for all other methods. b) Reduction to 15 dimensions followed by UMAP visualization for the Zheng 8eq dataset. glmpca_nb, glmpca_poi: GLM-PCA with negative binomial and Poisson likelihoods. pca_log: PCA on log-CPM. pca_rd, pca_rp: PCA on approximate multinomial deviance and Pearson residuals.

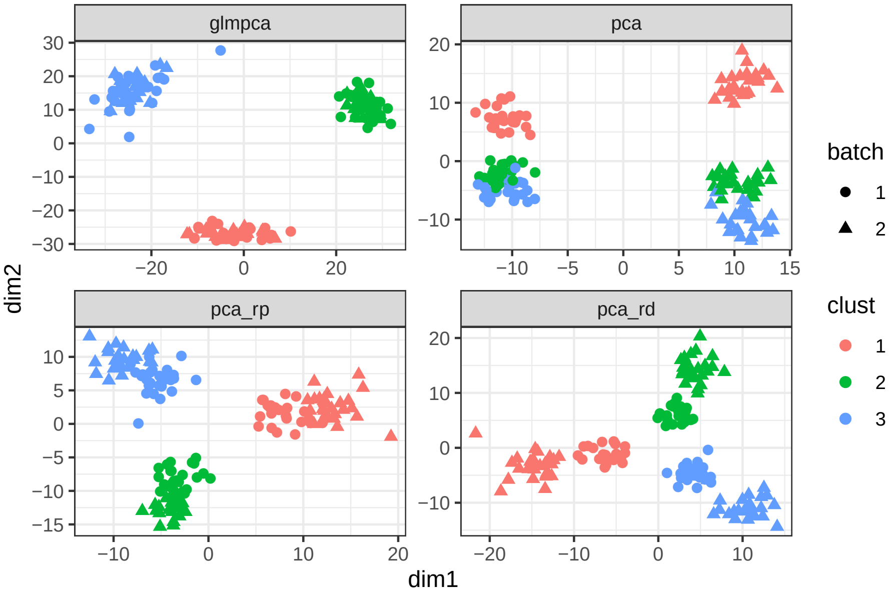

Figure S4: Deviance and Pearson residual approximations to GLM-PCA outperform PCA in the presence of a simulated strong batch effect. Batch 1 (circles) had total counts of 1,000 while Batch 2 (triangles) had total counts of 2,000. Biological clusters (colors) are poorly identified by PCA on log-CPM since the first PC was aligned with the batch effect. GLM-PCA completely removed the batch effect, while both residual approximations partially removed the batch effect. pca_rd: PCA on deviance residuals, pca_rp: PCA on Pearson residuals.

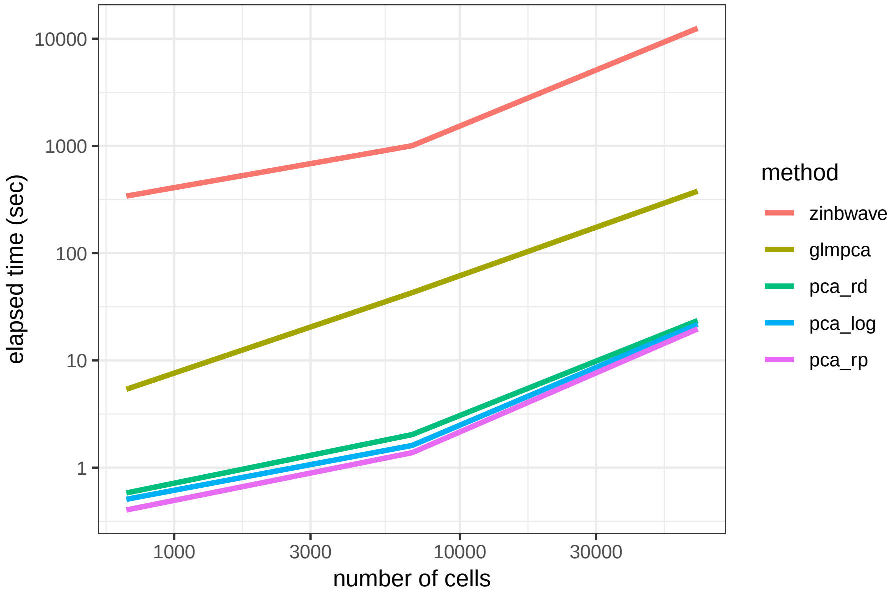

Figure S5: Computational speed comparison of dimension reduction methods Poisson GLM-PCA (glmpca), ZINB-WAVE (zinbwave), PCA on deviance residuals (pca_rd), PCA on Pearson residuals (pca_rp), and PCA on log-CPM (pca_log).

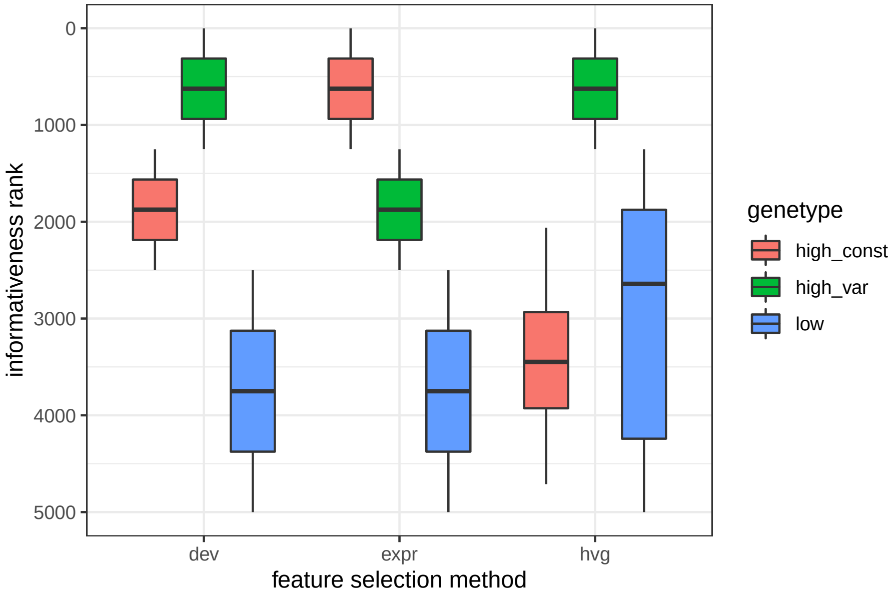

Figure S6: Feature selection by deviance identifies high and variably expressed genes, while highly expressed gene filtering identifies high and constant expressed genes in simulated data. A low rank indicates the criterion found the gene to be highly informative. dev: high deviance genes, expr: highly expressed genes, hvg: highly variable genes. high_const: high and constantly expressed, high_var: high and variably expressed.

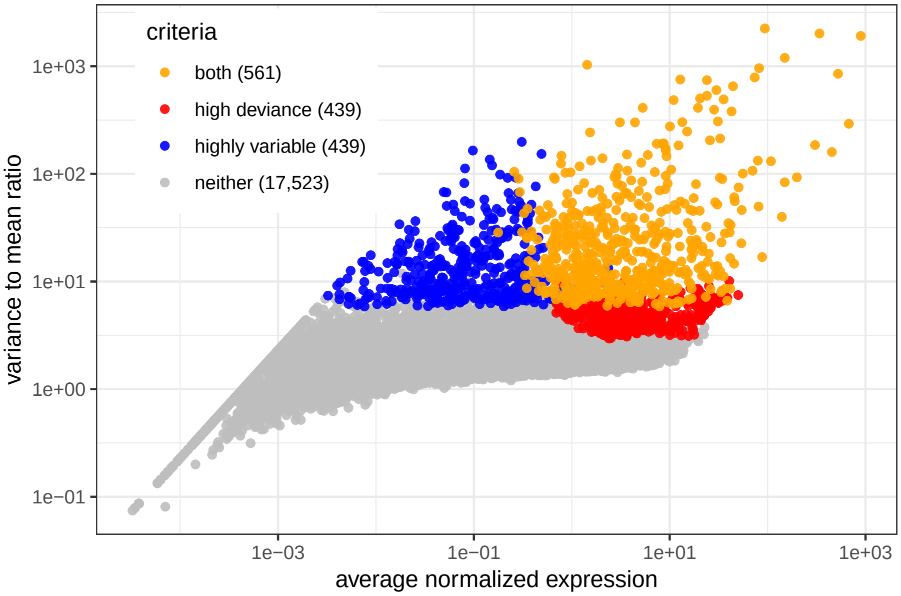

Figure S7: Comparison of top 1,000 genes selected as most informative in the Muraro dataset. The variance to mean ratio for each gene is plotted against the average expression. Counts were normalized using scran. Colors represent genes that are in the top 1,000 ranked by variability (blue, orange) and top 1,000 ranked by approximate multinomial deviance (red, orange). Orange indicates genes identified by both criteria, while gray indicates genes identified by neither criteria. Note that highly expressed genes have large values on the horizontal axis. The number of genes in each category is shown in parentheses.

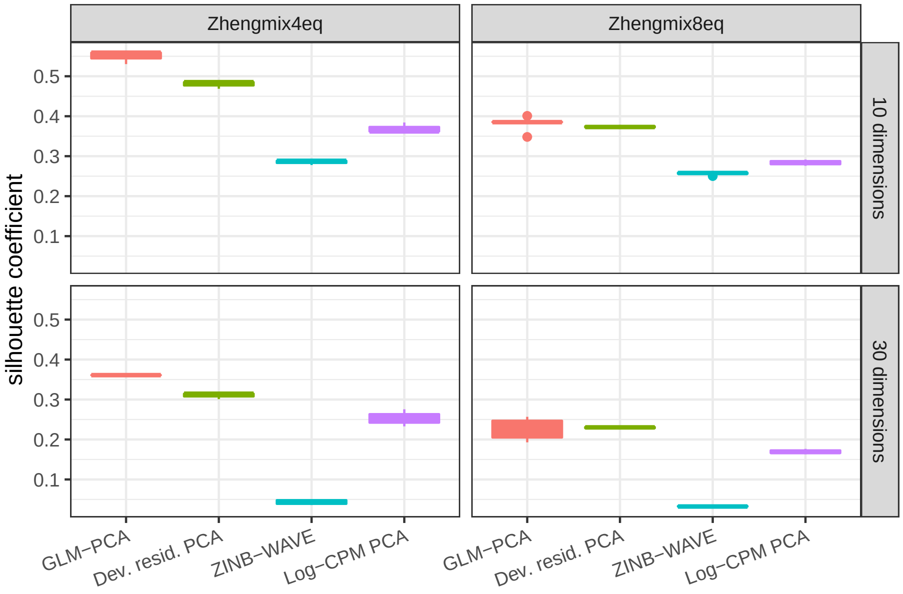

(a) Dimension reduction

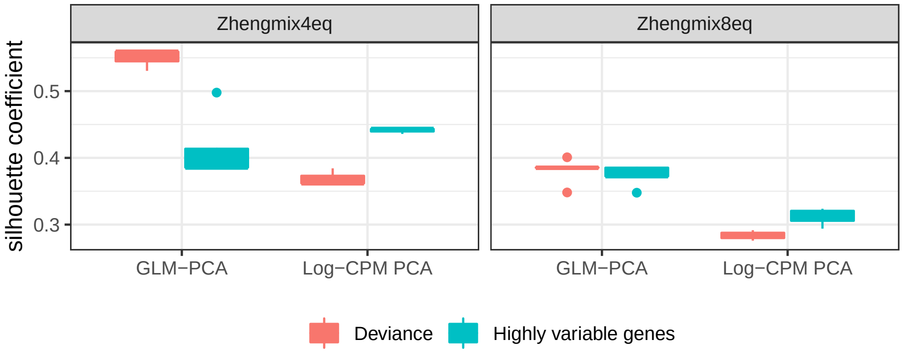

(b) Feature selection

Figure S8: Dimension reduction with GLM-PCA and feature selection using deviance improves Seurat clustering performance as measured by silhouette coefficient (larger=better). Each column represents a different ground-truth dataset from Duo et al. a) Comparison of dimension reduction methods based on the top 1,500 informative genes identified by approximate multinomial deviance. The Poisson approximation to the multinomial was used for GLM-PCA. Dev. resid. PCA: PCA on approximate multinomial deviance residuals. b) Comparison of feature selection methods. The top 1,500 genes identified by deviance and highly variable genes were passed to two different dimension reduction methods: GLM-PCA and PCA on log transformed CPM. Only results with the number of clusters within 25% of the true number are presented.

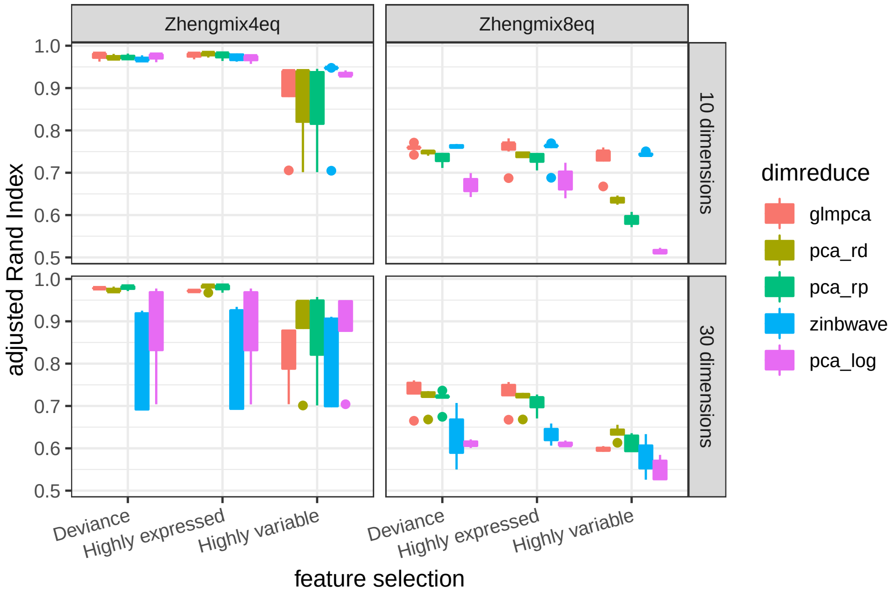

Figure S9: Comparison of Seurat clustering performance for all dimension reduction and feature selection methods on ground-truth datasets from Duo et al as measured by adjusted Rand index (larger=better). The number of informative genes was fixed at 1,500. The Poisson approximation to the multinomial was used for GLM-PCA. Only results with the number of clusters within 25% of the true number are presented. Abbreviations: dimreduce: dimension reduction method, pca_rd: PCA on deviance residuals, pca_rp: PCA on Pearson residuals, pca_log: PCA on log-CPM.

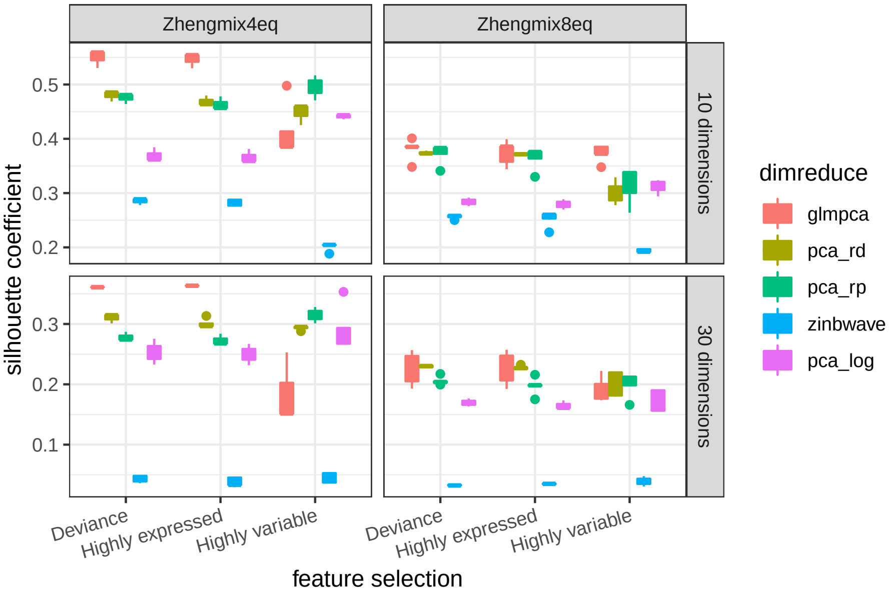

Figure S10: Comparison of Seurat clustering performance for all dimension reduction and feature selection methods on ground-truth datasets from Duo et al as measured by silhouette coefficient (larger=better). The number of informative genes was fixed at 1,500. The Poisson approximation to the multinomial was used for GLM-PCA. Only results with the number of clusters within 25% of the true number are presented. Abbreviations: dimreduce: dimension reduction method, pca_rd: PCA on deviance residuals, pca_rp: PCA on Pearson residuals, pca_log: PCA on log-CPM.

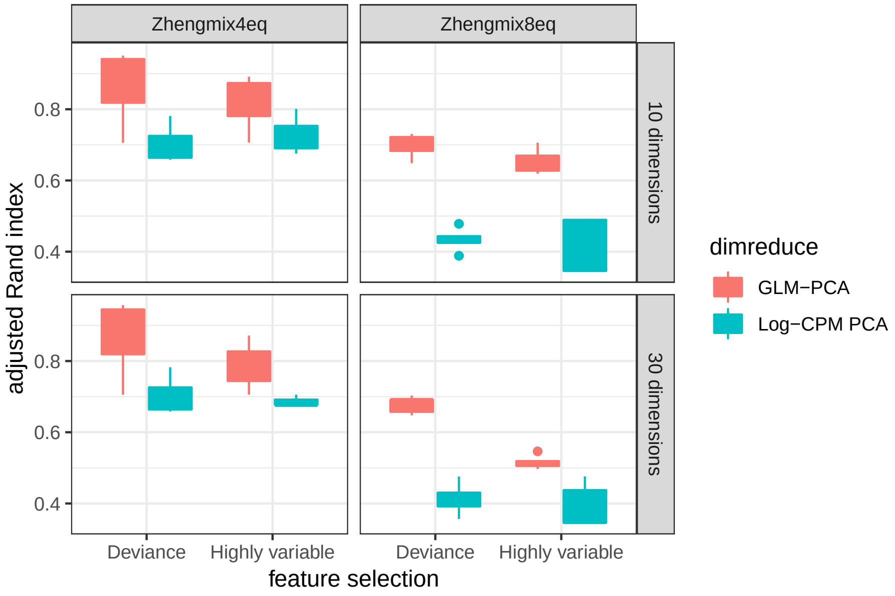

Figure S11: Dimension reduction with GLM-PCA and feature selection using deviance improves k-means clustering performance as measured by adjusted Rand index (larger=better). Each column represents a different ground-truth dataset from Duo et al. The top 1,500 informative genes were identified by approximate multinomial deviance and highly variable genes. The Poisson approximation to the multinomial was used for GLM-PCA. Only results with the number of clusters within 25% of the true number are presented.

Table S1: High deviance genes overlap with highly variable genes but not highly expressed genes in simulated data. Same data as Figure S6. High and low indicate whether or not the gene was flagged as in the top 1,000 most informative by each criterion. hvg: highly variable genes, dev: high deviance genes.

(a) not highly expressed

	**hvg low**	**hvg high**
**dev low**	2,918	82
**dev high**	82	918

(b) highly expressed

	**hvg low**	**hvg high**
**dev low**	1,000	0
**dev high**	0	0

Table S2: Deviance identifies informative genes not found by other methods. Same data as Figure S7. High and low indicate whether or not the gene was flagged as in the top 1,000 most informative by each criterion. hvg: highly variable genes, dev: high deviance genes.

(a) not highly expressed

	**hvg low**	**hvg high**
**dev low**	16,890	439
**dev high**	239	394

(b) highly expressed

	**hvg low**	**hvg high**
**dev low**	633	0
**dev high**	200	167

Table S3: GLM-PCA has best clustering performance on Haber dataset. Feature selection used 2,000 informative genes. Clustering was by Seurat's Louvain algorithm with resolution 1.8. Accuracy was assessed by comparing to original authors' cluster label annotations. ARI: adjusted Rand index.

**dimension reduction**	**feature selection**	**ARI**	**silhouette**
Poisson GLM-PCA	deviance	0.442	0.195
negative binomial GLM-PCA	deviance	0.428	0.202
PCA on log-CPM	highly variable genes	0.385	0.174
ZINB-WAVE	highly variable genes	0.372	0.120
PCA on deviance residuals	deviance	0.357	0.142
PCA on Pearson residuals	deviance	0.353	0.147

Table S4: GLM-PCA has best clustering performance on Muraro dataset. Feature selection used 2,000 informative genes. Clustering was by Seurat's Louvain algorithm with resolution 0.8. Accuracy was assessed by comparing to original authors' cluster label annotations. ARI: adjusted Rand index.

**dimension reduction**	**feature selection**	**ARI**	**silhouette**
Poisson GLM-PCA	deviance	0.664	0.274
ZINB-WAVE	highly variable genes	0.596	0.240
negative binomial GLM-PCA	deviance	0.586	0.300
PCA on log-CPM	highly variable genes	0.515	0.305
PCA on deviance residuals	deviance	0.514	0.218
PCA on Pearson residuals	deviance	0.499	0.218
