# Supplementary material for: Feature selection and dimension reduction for single-cell RNA-Seq based on a multinomial model
Source: Genome Biol. 2019 Dec 23;20:295. doi: 10.1186/s13059-019-1861-6 (PMC6927135; doi:10.1186/s13059-019-1861-6)
Supplement: Supplementary file 2 — Additional file 2 Review history. [file 13059_2019_1861_MOESM2_ESM.docx]

Review History

**First round of review**

**Reviewer 1**

**Were you able to assess all statistics in the manuscript, including the appropriateness of statistical tests used?**

Yes: All statistics presented in the data are valid and appropriate.

**Were you able to directly test the methods?**

No

**Comments to author:**

The manuscript by Townes et al. addresses the challenge of variability in single-cell RNA-seq data associated with dropout events, i.e. the absence of sequenced transcripts for a gene associated with sampling noise and potentially other sources of variability. Using five different ground truth datasets with well-defined sources of technical and/or biological variability they demonstrate that the observed dropout rates, at least for these datasets generated with droplet-based microfluidic techniques, are consistent with multinomial sampling and do not require modeling zero inflation. They further show that log-transformation after addition of arbitrary pseudocounts, a common approach in single-cell transcriptome analysis, leads to an exaggerated separation of cells with zero and non-zero counts, and frequently may explain the first principal component in a conventional PCA.

As an alternative dimensional reduction scheme that appropriately accounts for zero-counts as a consequence of multinomial sampling, the authors propose a GLM-PCA which adjusts the conventional PCA relying on normally distributed data to accommodate (overdispersed) count data following a Poisson or negative binomial distribution by introducing such distributions into the objective function. They show that this approach successfully eliminates variability associated with cell-to-cell variation in total UMI counts (implying dropout-associated variability) and increases clustering performance on a two ground-truth datasets compared to other dimensional reduction schemes. They also demonstrate that a conventional PCA on multinomial Pearson residual represents a fast alternative to the full GLM-PCA.

Overall, the work is technically solid and the authors provide a mathematical foundation to common assumptions and approaches in single-cell RNA-seq analysis. In this language they nicely demonstrate the consistency of zero-counts in UMI-based single-cell RNA-seq data with a multinomial distribution. However, this has also been recognized previously and is per se not an unexpected finding.

Although their GLM-PCA method is potentially useful they have not sufficiently demonstrated its performance as discussed below. Another problem is that this method is not a gene-level normalization but instead introduces latent factors (principle components) which limits its scope and does not permit, e.g., the direct derivation of differentially expressed genes from the corrected data and other gene-level analyses.

I have a number of concerns that need to be addressed by the authors:

1. While the authors demonstrate successfully that the technical noise of lowly expressed genes follows a Poisson (or multinomial) distribution, they fail to test this for highly expressed genes. It has been previously described that, at higher expression, global cell-to-cell variability in sequencing efficiency, i.e. cell-to-cell variability affecting all genes equally, becomes the more dominant source of technical noise, and this noise component leads to overdispersion, thus requiring a negative binomial distribution for appropriate modeling (Grün et al., Nature Methods, 2014). If the authors claim that a Poisson model is sufficient to explain the data they should investigate if this additional technical noise may dominate the GLM-PCA. This could be addressed by simulations, varying the relative contribution of this noise component. The fact that high-deviance genes strongly overlap with highly expressed genes could be a consequence of this noise component.

2. They argue that GLM-PCA with a negative binomial distribution becomes unstable due to the difficulty of estimating the dispersion parameter. This could potentially be solved by an appropriate regularization.

3. The clustering benchmarking of the various dimensional reduction and feature selection methods is limited and not very convincing. (1) The authors should not only test the Rand index but at least also analyze cluster separation, e.g. by computing the shilouette. (2) They should in silico perturb the data to investigate how well their method deals with technical variability in total UMI counts per cells. For instance, they could split their dataset and sample only a fraction of sequencing reads for one subset and then co-analyze the subsets. (3) They should visualize the clustering results for all methods in a dimensional-reduction representation. (4) It also seems that GLM-PCA outperforms the other methods only for 30 dimensions. To make a more general statement, the authors should analyze the performance as a function of the dimensions. It was also not clear to me by which criterion the number of dimensions was selected. Finally, they should compare the methods on a more complex datasets with higher sub-type variety. The intestinal data from Haber et al., Nature, 2017 represent a good test case, in particular, the taxonomy of enteroendocrine cells shown in Figure 3 of this work.

4. They argue that conventional PCA often ends up identifying sequencing depth-related variability as the major principle component. They should provide a more thorough analysis for their test datasets to make this point, i.e. compare the gene loadings for the first X principle components of the conventional PCA and the GLM-PCA.

5. Trajectory inference is an important application of single-cell RNA-seq studies, which requires the detection of continuous manifolds in the data. It is important to demonstrate that the proposed GLM-PCA does not compromise the ability to infer trajectories. This could be addressed by, e.g., applying the method to bone-marrow progenitor data (e.g. Dahlin et al., 2018, Blood) and inferring trajectories on these data with established approaches (PAGA, diffusion maps, Monocle3 etc. )

6. The method was only tested for droplet-based data. The authors should demonstrate the performance also for plate-based UMI protocols, which represent a powerful class of protocols with on average higher sensitivity than droplet-based methods. For instance, they could apply it to the CEL-Seq2 pancreas data from Muraro et al., 2016, Cell Systems.

7. To make their method readily available and ensure usability for a broader community the authors should provide an R package for their method (CRAN or Bioconductor).

8. The authors should also correct some of their general statements and references of the past literature. For example, in the introduction they write that "Early scRNA-Seq studies using protocols such as SMART-Seq2[8] analyzed these read counts directly, and several methods were developed to facilitate this [9]. However, newer protocols typically include unique molecular identiers (UMIs) which enable computational removal of PCR duplicates [10], producing UMI counts." However, SMART-Seq was developed in parallel with STRT-seq (Islam et al., 2014, Nature Methods) and CEL-seq which integrated UMIs early on (Grün et al., 2014, Nature Methods). It is was not so much a question of method progress but rather of the purpose of the method. SMART-Seq2 is still the state-of-the-art method for full-length sequencing, which cannot be achieved by UMI-based protocols.

9. Central results of the study, e.g. that UMI-based single-cell RNA-seq data follow a negative binomial and the interpretation of the different sources of technical noise were described already in 2014 (Grün et al., 2014, Nature Methods), but this reference is missing in the manuscript.

**Reviewer 2**

**Were you able to assess all statistics in the manuscript, including the appropriateness of statistical tests used?**

Yes: method paper

**Were you able to directly test the methods?**

No

**Comments to author:**

\###

Review of Feature Selection and Dimension Reduction for Single Cell RNA-Seq based on a Multinomial Model

Summary:

The authors present an exploration of statistical models for UMI-based scRNAseq data and propose to make connection to the biochemical processes that underly the data generation without trying to build a bottom-up model. This view on the topic is appropriate in terms of complexity and much appreciated. The manuscript is divided in small sections with fairly clear and actionable statements which is again well appreciated. However, the evidence for the statements in the individual sections is somewhat weak in places; this is IMHO quite damaging to the manuscript see below. Please consider my suggestions below to strengthen the points that you want to make. I am sure this manuscript could provide a useful contribution to the field if these points are tackled.

Statement on impact:

Many points related to noise models that were made in the manuscript have been made before from a potentially more empirical setting but it has been difficult to coherently argue for one noise model. This manuscript could contribute significantly to this discussion. The points on GLM-PCA are novel.

Main contributions:

- multinomial noise describes UMI-based scRNAseq data

An important point that is core to the manuscript but in its current form not well enough supported by analyses.

- GLM-PCA outperforms PCA because the data is not normal distributed

A nice idea that extends noise models beyond differential expression (their major "use case"). Please discuss accuracy of the proposed PCA based on residuals.

- feature selection based on deviance statistic

The points made in favor of this suggestion are weak, I would consider removing this from the manuscript if the authors cannot strongly improve this (comments 6, 7.2).

Major comments:

- 1: 2.2 UMI count distribution differs from reads

The authors make the point that UMI data is not zero-inflated. This notion is nowadays widely believed to be true in the field but has not been well supported by published studies so that there is still discussions about this. Exploring this issue on UMI data that has not been deduplciated is a very good idea. I would ask the authors to really put more effort into quantifying this effect though. This statement is about choice of noise model which has vast effects on differential expression analysis and p-values, any hypothesis presented in this context should be backed by more solid analysis. Right now, the authors present histograms of a single gene, this is clearly not sufficient. I do agree with the authors that ZI is likely a PCR-artefact, but finding evidence for this has been an issue, which is why other studies has stayed clear of touching this issue. This is an important point to make and a good contribution of the manuscript so I would really encourage the authors to work on this. Some ideas, but please do not let this constrain your creativity: model selection based on likelihood-based model fits, moment-based statistics (e.g. number of zeros versus mean)..

- 2: 2.3 Multinomial sampling distribution for UMI counts

-- 2.1 You state that a multinomial distribution can be approximated by independent poisson and negative binomial distribtions. This is an important point as negative binomial noise is a common choice for modelling RNA-seq and scRNA-seq data. As this is a distribution centric paper, it would be nice to supply some details on how well this approximations work, ie at what number of samples they break down etc.

-- 2.1 You base the main point of this section on the argument that the multinomial fits two characteristics of the data:

* fraction of zeros is inversely related to total number of UMIs

* the probability of zero counts is a decreasing function of the mean

--- 2.1.1 Both arguments are related to first moments of the distribution per gene or across the sample. I am not sure how well they would serve to distinguish noise models

as the major distinction between unimodal noise models lies in the second and higher order moments.

--- 2.1.2 More importantly, you do not compare these distributions against other noise models. This is a weakness of this analysis. It is hard to argue that one should use multinomial noise models or their approximations because they fit to some more or less arbitrarily selected summary statistics. I would suggest to compare to other noise models (normal, log-normal, hurdle etc.). Again likelihood-based model selection may be an option to select between models.

- 3: 2.4 Normalisation and log-transformation distorts UMI data

-- 3.1 You argue that artefacts occur in dimension reduction by showing that the first PC of log transformed data is correlated with fraction of zeros. You however fail to show whether this also occurs in the non-log-transformed control of your analysis that you should have done. Can you please add this control analysis?

-- 3.2 The major reason for log-transformation is a feature engineering perspective on the data: It reduces the difference in variance contributed by each gene that is related to its mean (mean-variance relation ship of count distributions) and thereby reduces the bias of embeddings and clusterings to depend mostly on highly expressed genes. It would be important to discuss this.

- 4: 2.5 Zero inflation is an artifact of log-normalization

-- 4.1 The statement made here is relatively straight-forward but not wrong. You may take it out if you are pressured for space.

- 5: 2.6 Generalized PCA for dimension reduction of sparse counts, 2.7 Deviance residuals provide fast approximation to GLM- PCA, 2.10 Computational efficiency of multinomial models

-- 5.1 The points made here is nice, again it crucially depends on the negative control analysis that I suggest in comment 3.1.

-- 5.2 Scalability and run time are clearly important issues for PCA as PCA is often used as a reduction before kNN-graph based methods or other dimension reductions are used. Please add 2.10 closer or into 2.6, I was confused by not finding it when reading this section.

-- 5.3 Approximation of GLM-PCA by PCA on deviance residuals: The concept is clear here, but clearly the remainder of the optimisation is potentially biased as the the wrong noise model is assumed in the vanilla PCA implementation that is used after the feature space is transformed to residuals. It would be important to discuss accuracy with respect to PCA and GLM-PCA of this residual-based PCA, for example in simulation studies.

- 6: 2.8 Feature selection using deviance

- The intuition why this would work is clear. However, I find that the motivation of how it is working better is lacking, if you take expression strength as a gold standard, why not use expression strength in the first place in practice?

- 7: 2.9 Multinomial models improve unsupervised clustering

-	7.1 The argument presented here is based on k-means in the latent space. I would like to really emphasize that k-means in the latent space is not common practice for scRNA-seq, almost every large study presents clusterings from the louvain/leiden method. Please benchmark on this, otherwise you may risk to appear to cherry-pick the benchmarking scenario.

-	7.2 You again make the point that feature selection on deviance performs similarly to total expression.

Minor comments:

- "(often > 90% of the data, sometimes called dropouts)." I would avoid the use of drop-out unless you discuss that this is not drop-out in the zero-inflated sense right at this point. The fact that dropout is used for the occurrence of a zero in a simple sampling process (such as multinomial as suggested here) is a major confusion in the field and we can only rid ourselves from this confusion by coherently avoiding or redefining it at every occurrence.

- Could you state the number of PCR cycles for library amplification used in the most commom protocols? This would be helpful to guide intuition as to how large the effects can be here.

- "The statistical approaches described here have not been validated against scRNA-Seq data without UMIs, such as SMART-Seq2 and other plate protocols [8], since non-UMI data contain PCR duplicates. To apply the ideas to these data, one would need to be able to infer the UMI counts for data with PCR replicates [7]." I do not see the point of this section and would remove it. Clearly, all your statements are about UMI data.

**Authors Response**

**Point-by-point responses to the reviewers’ comments:**

*Reviewer #1: The manuscript by Townes et al. addresses the challenge of variability in single-cell RNA-seq data associated with dropout events, i.e. the absence of sequenced transcripts for a gene associated with sampling noise and potentially other sources of variability. Using five different ground truth datasets with well-defined sources of technical and/or biological variability they demonstrate that the observed dropout rates, at least for these datasets generated with droplet-based microfluidic techniques, are consistent with multinomial sampling and do not require modeling zero inflation. They further show that log-transformation after addition of arbitrary pseudocounts, a common approach in single-cell transcriptome analysis, leads to an exaggerated separation of cells with zero and non-zero counts, and frequently may explain the first principal component in a conventional PCA.*

*As an alternative dimensional reduction scheme that appropriately accounts for zero-counts as a consequence of multinomial sampling, the authors propose a GLM-PCA which adjusts the conventional PCA relying on normally distributed data to accommodate (overdispersed) count data following a Poisson or negative binomial distribution by introducing such distributions into the objective function. They show that this approach successfully eliminates variability associated with cell-to-cell variation in total UMI counts (implying dropout-associated variability) and increases clustering performance on a two ground-truth datasets compared to other dimensional reduction schemes. They also demonstrate that a conventional PCA on multinomial Pearson residual represents a fast alternative to the full GLM-PCA.*

*Overall, the work is technically solid and the authors provide a mathematical foundation to common assumptions and approaches in single-cell RNA-seq analysis. In this language they nicely demonstrate the consistency of zero-counts in UMI-based single-cell RNA-seq data with a multinomial distribution. However, this has also been recognized previously and is per se not an unexpected finding.*

*Although their GLM-PCA method is potentially useful they have not sufficiently demonstrated its performance as discussed below. Another problem is that this method is not a gene-level normalization but instead introduces latent factors (principle components) which limits its scope and does not permit, e.g., the direct derivation of differentially expressed genes from the corrected data and other gene-level analyses.*

*I have a number of concerns that need to be addressed by the authors:*

*1. While the authors demonstrate successfully that the technical noise of lowly expressed genes follows a Poisson (or multinomial) distribution, they fail to test this for highly expressed genes. It has been previously described that, at higher expression, global cell-to-cell variability in sequencing efficiency, i.e. cell-to-cell variability affecting all genes equally, becomes the more dominant source of technical noise, and this noise component leads to overdispersion, thus requiring a negative binomial distribution for appropriate modeling (Grün et al., Nature Methods, 2014). If the authors claim that a Poisson model is sufficient to explain the data they should investigate if this additional technical noise may dominate the GLM-PCA. This could be addressed by simulations, varying the relative contribution of this noise component. The fact that high-deviance genes strongly overlap with highly expressed genes could be a consequence of this noise component.*

***We now include a second biological negative control from Tung et al (a plate-based protocol), from which we examined 5,000 random genes with both UMI and read counts. In the Results and discussion Section 2.3, second to last paragraph, we describe an assessment of the goodness-of-fit of seven null distributions (including Poisson and negative binomial) to both the Tung et al. and Zheng et al. data (Figure S2). For UMI count data, we demonstrate that the multinomial, Dirichlet-multinomial, and Poisson (as approximation to multinomial) distributions fit best. When applied to read counts, the zero-inflated lognormal was the best fitting distribution followed by the Dirichlet-multinomial.***

***Furthermore, as the reviewer suggests, negative binomial is generated marginally if the size factors representing global sequencing depth and/or capture efficiency in the Poisson model follow a gamma distribution. A nice illustration (via simulation) is provided by Svensson:*** [***https://www.nxn.se/valent/2018/1/30/count-depth-variation-makes-poisson-scrna-seq-data-negative-binomial***](https://www.nxn.se/valent/2018/1/30/count-depth-variation-makes-poisson-scrna-seq-data-negative-binomial)***. So, if we condition on these size factors (GLM-PCA does this by default) by including them as offsets in a Poisson model, it removes the cell-to-cell variability so the extra dispersion of negative binomial is no longer necessary. Of course, this assumes the extra variability is constant across genes, which may not always be the case in experiment generated data. In such cases the negative binomial model may be appropriate, as it can be considered an approximation to the Dirichlet-multinomial as we note in the methods.***

*2. They argue that GLM-PCA with a negative binomial distribution becomes unstable due to the difficulty of estimating the dispersion parameter. This could potentially be solved by an appropriate regularization.*

***We have now implemented negative binomial GLM-PCA with a lognormal prior as regularization. This prior weakly shrinks the shape parameter toward one (geometric distribution). For example, in Figure S3 we show UMAP visualizations of Poisson and negative binomial GLM-PCA. In our assessments, we found the difference between using GLM-PCA with the Poisson and negative binomial distribution is small.***

*3. The clustering benchmarking of the various dimensional reduction and feature selection methods is limited and not very convincing.*

*(1) The authors should not only test the Rand index but at least also analyze cluster separation, e.g. by computing the shilouette.*

***We now include silhouette coefficients for all benchmarking results, see results 2.10 and Figures S7,S9. We found results consistent to the results reported in the first version of the manuscript which used Adjusted Rand Index: GLM-PCA outperforms the other dimensionality reduction approaches.***

*(2) They should in silico perturb the data to investigate how well their method deals with technical variability in total UMI counts per cells. For instance, they could split their dataset and sample only a fraction of sequencing reads for one subset and then co-analyze the subsets.*

***We have now performed this analysis (see figure below). Here are the data visualizations of the reduced dimensions for the Zheng4eq dataset before and after downsampling a random 50% of the cells to the equal the total counts of the smallest cell. In addition, we note that there is variation in the total UMI counts across cells in all the datasets considered, albeit not in the discrete manner proposed here.***

*(3) They should visualize the clustering results for all methods in a dimensional-reduction representation.*

***We now include dimension reduction visualizations for Zheng 4eq and Zheng 8eq dataset in Figure S3 and discussion at the end of results 2.6***

*(4) It also seems that GLM-PCA outperforms the other methods only for 30 dimensions. To make a more general statement, the authors should analyze the performance as a function of the dimensions. It was also not clear to me by which criterion the number of dimensions was selected.*

***While all methods had high accuracy with L=10 on the Zheng4eq dataset, PCA had worse accuracy with L=10 on the Zheng8eq dataset, which is harder to cluster because it contains multiple subtypes of T cells. Even in situations in which accuracy is the same, we now show using silhouette (see response to point #3.1 above) that GLM-PCA and its residuals approximations outperform in terms of cluster separation. We chose the L=10 and L=30 dimensions because we wanted our assessments to be comparable to those of Duo et al 2018.***

*Finally, they should compare the methods on a more complex datasets with higher sub-type variety. The intestinal data from Haber et al., Nature, 2017 represent a good test case, in particular, the taxonomy of enteroendocrine cells shown in Figure 3 of this work.*

***We now include Table S1 summarizing clustering performance of competing methods when applied to this dataset, with discussion at the end of results 2.10. Using the Haber et al. (Table S1) data set, GLM-PCA with the Poisson model and negative binomial model reported the largest Adjusted Rand Index.***

*4. They argue that conventional PCA often ends up identifying sequencing depth-related variability as the major principle component. They should provide a more thorough analysis for their test datasets to make this point, i.e. compare the gene loadings for the first X principle components of the conventional PCA and the GLM-PCA.*

***We are happy to perform an analys that the reviewer is describing, but we are currently unclear on the specific details for an analysis that is being proposed. For example, we are unclear about how the loadings would inform us about sequencing depth variability, which is a cell-specific quantity not a gene-specific one. Hypothetically, let’s assume we applied PCA and GLM-PCA each with 2 dimensions to a given dataset with 2,000 informative genes and obtained the loadings matrices (each matrix of dimension of 2,000 genes x 2 reduced dimensions). Based on our understanding the proposed analysis, is the reviewer asking to look at the top 5 genes with the largest loading value for each dimension for each method? While this might be informative, it would not be informative for a cell-specific quantity of sequencing depth/ total UMIs per cell. Could the reviewer provide more details on the proposed analysis?***

*5. Trajectory inference is an important application of single-cell RNA-seq studies, which requires the detection of continuous manifolds in the data. It is important to demonstrate that the proposed GLM-PCA does not compromise the ability to infer trajectories. This could be addressed by, e.g., applying the method to bone-marrow progenitor data (e.g. Dahlin et al., 2018, Blood) and inferring trajectories on these data with established approaches (PAGA, diffusion maps, Monocle3 etc. )*

***We avoided trajectory inference because of the difficulty in establishing a ground truth to benchmark against. We would prefer to leave this out of the results and mention trajectory analysis along with spatial and differential expression in the conclusion section as areas of scRNA-Seq for others to extend the multinomial concepts in future studies. Nevertheless please see below for two visualizations of the Dahlin data. Here, we only include sample SIGAB1 which contains LSK cells, meaning it is enriched for the progenitor cells and less abundant in the differentiated cells (Dahlin figure 1c). The PCA plot is on log2(1+CPM) and uses the top 2,000 highly variable genes. The GLM-PCA plot is on the top 2,000 high deviance genes. We highlight in color cells for which the expression of marker genes indicated by Dahlin was high.***

*6. The method was only tested for droplet-based data. The authors should demonstrate the performance also for plate-based UMI protocols, which represent a powerful class of protocols with on average higher sensitivity than droplet-based methods. For instance, they could apply it to the CEL-Seq2 pancreas data from Muraro et al., 2016, Cell Systems.*

***We now include this analysis in results 2.10 last paragraph, and in Table S2. Using the Muraro et al. (Table S2), GLM-PCA with the Poisson model reported the largest Adjusted Rand Index.***

*7. To make their method readily available and ensure usability for a broader community the authors should provide an R package for their method (CRAN or Bioconductor).*

***We have created an R package publicly available at*** [***https://github.com/willtownes/glmpca***](https://github.com/willtownes/glmpca) ***and vignette (https://github.com/willtownes/glmpca/blob/master/vignettes/glmpca.Rmd ). We are working to improve the documentation before submitting to CRAN in the near future. We updated the Declarations section to include the URL.***

*8. The authors should also correct some of their general statements and references of the past literature. For example, in the introduction they write that "Early scRNA-Seq studies using protocols such as SMART-Seq2[8] analyzed these read counts directly, and several methods were developed to facilitate this [9]. However, newer protocols typically include unique molecular identiers (UMIs) which enable computational removal of PCR duplicates [10], producing UMI counts." However, SMART-Seq was developed in parallel with STRT-seq (Islam et al., 2014, Nature Methods) and CEL-seq which integrated UMIs early on (Grün et al., 2014, Nature Methods). It is was not so much a question of method progress but rather of the purpose of the method. SMART-Seq2 is still the state-of-the-art method for full-length sequencing, which cannot be achieved by UMI-based protocols.*

***We have modified the first paragraph of the introduction to reflect these suggestions. We now include a citation of Grun et al. (2014) where before we only cited Islam et al. (2014).***

*9. Central results of the study, e.g. that UMI-based single-cell RNA-seq data follow a negative binomial and the interpretation of the different sources of technical noise were described already in 2014 (Grün et al., 2014, Nature Methods), but this reference is missing in the manuscript.*

***We now include the Grun et al. (2014) reference.***

*Reviewer #2: ###*

*Review of Feature Selection and Dimension Reduction for Single Cell RNA-Seq based on a Multinomial Model*

*Summary:*

*The authors present an exploration of statistical models for UMI-based scRNAseq data and propose to make connection to the biochemical processes that underly the data generation without trying to build a bottom-up model. This view on the topic is appropriate in terms of complexity and much appreciated. The manuscript is divided in small sections with fairly clear and actionable statements which is again well appreciated. However, the evidence for the statements in the individual sections is somewhat weak in places; this is IMHO quite damaging to the manuscript see below. Please consider my suggestions below to strengthen the points that you want to make. I am sure this manuscript could provide a useful contribution to the field if these points are tackled.*

*Statement on impact:*

*Many points related to noise models that were made in the manuscript have been made before from a potentially more empirical setting but it has been difficult to coherently argue for one noise model. This manuscript could contribute significantly to this discussion. The points on GLM-PCA are novel.*

*Main contributions:*

*- multinomial noise describes UMI-based scRNAseq data*

*An important point that is core to the manuscript but in its current form not well enough supported by analyses.*

*- GLM-PCA outperforms PCA because the data is not normal distributed*

*A nice idea that extends noise models beyond differential expression (their major "use case"). Please discuss accuracy of the proposed PCA based on residuals.*

***We discuss the accuracy of PCA on deviance and Pearson residuals in the Results and discussion Section 2.10. These points are also included in additional assessments, see for example Figures 5a,S3,S8 and Tables S1 and S2.***

*- feature selection based on deviance statistic*

*The points made in favor of this suggestion are weak, I would consider removing this from the manuscript if the authors cannot strongly improve this (comments 6, 7.2).*

***In Figure 5b, we show that GLM-PCA with deviance feature selection outperforms GLM-PCA with highly variable genes feature selection when assessing the Adjusted Rand Index. We address deviance versus highly expressed genes below.***

*Major comments:*

*- 1: 2.2 UMI count distribution differs from reads*

*The authors make the point that UMI data is not zero-inflated. This notion is nowadays widely believed to be true in the field but has not been well supported by published studies so that there is still discussions about this. Exploring this issue on UMI data that has not been deduplicated is a very good idea. I would ask the authors to really put more effort into quantifying this effect though. This statement is about choice of noise model which has vast effects on differential expression analysis and p-values, any hypothesis presented in this context should be backed by more solid analysis. Right now, the authors present histograms of a single gene, this is clearly not sufficient. I do agree with the authors that ZI is likely a PCR-artefact, but finding evidence for this has been an issue, which is why other studies has stayed clear of touching this issue. This is an important point to make and a good contribution of the manuscript so I would really encourage the authors to work on this. Some ideas, but please do not let this constrain your creativity: model selection based on likelihood-based model fits, moment-based statistics (e.g. number of zeros versus mean)..*

***We now provide Figure S2 (and discussion in Results and discussion Section 2.3) where we compare goodness-of-fit of 7 different possible distributions (including some zero inflated) to both UMI counts and read counts of the same data. Here, we demonstrate that the multinomial, Dirichlet-multinomial, and Poisson (as approximation to multinomial) distributions fit best. When applied to read counts, the zero-inflated lognormal was the best fitting distribution followed by the Dirichlet-multinomial.***

*- 2: 2.3 Multinomial sampling distribution for UMI counts*

*-- 2.1 You state that a multinomial distribution can be approximated by independent poisson and negative binomial distribtions. This is an important point as negative binomial noise is a common choice for modelling RNA-seq and scRNA-seq data. As this is a distribution centric paper, it would be nice to supply some details on how well this approximations work, ie at what number of samples they break down etc.*

***We discuss this in Methods 4.1, the paragraph beginning “First, note that if correlation is ignored…”. The approximation breaks down when the total counts of a cell are very small, when a single feature (gene) has a probability much larger than all the others, or when the number of features is small. We cite references from the statistical literature that rigorously quantify and put bounds on the approximation error. In addition, we now provide a more intuitive explanation in the manuscript.***

*-- 2.1 You base the main point of this section on the argument that the multinomial fits two characteristics of the data:*

** fraction of zeros is inversely related to total number of UMIs*

** the probability of zero counts is a decreasing function of the mean*

*--- 2.1.1 Both arguments are related to first moments of the distribution per gene or across the sample. I am not sure how well they would serve to distinguish noise models*

*as the major distinction between unimodal noise models lies in the second and higher order moments.*

***We now provide Figure S2 (and Results and discussion Section 2.3) to address this point. We have now expanded our analysis to assessing goodness-of-fit (using Bayesian information criterion or BIC) for 7 sampling distributions to each of the datasets containing biological replicate negative controls.***

*--- 2.1.2 More importantly, you do not compare these distributions against other noise models. This is a weakness of this analysis. It is hard to argue that one should use multinomial noise models or their approximations because they fit to some more or less arbitrarily selected summary statistics. I would suggest to compare to other noise models (normal, log-normal, hurdle etc.). Again likelihood-based model selection may be an option to select between models.*

***We now provide Figure S2 (and Results and discussion Section 2.3) to address this point.***

*- 3: 2.4 Normalisation and log-transformation distorts UMI data*

*-- 3.1 You argue that artefacts occur in dimension reduction by showing that the first PC of log transformed data is correlated with fraction of zeros. You however fail to show whether this also occurs in the non-log-transformed control of your analysis that you should have done. Can you please add this control analysis?*

***We now report Pearson correlation values for technical and biological (monocytes) replicates between PC1 and zero fraction both before and after log transformation of CPM values (Results and discussion Section 2.4 in the last paragraph). Note, log transforming reduces the correlation between PC1 and zero fraction. After centering and scaling, this is roughly equivalent to PCA on approximate multinomial Pearson residuals which we regard as an approximation to GLM-PCA.***

*-- 3.2 The major reason for log-transformation is a feature engineering perspective on the data: It reduces the difference in variance contributed by each gene that is related to its mean (mean-variance relation ship of count distributions) and thereby reduces the bias of embeddings and clusterings to depend mostly on highly expressed genes. It would be important to discuss this.*

***We had previously discussed the motivation for log transforming in the Methods Section 4.2 in that it facilitates interpreting differential expression in terms of fold changes. We now include the following in that section: “Also, for count data, the variance of each gene is a function of its mean, and log-transformation can help to prevent highly expressed outlier genes from overwhelming downstream analyses.”***

*- 4: 2.5 Zero inflation is an artifact of log-normalization*

*-- 4.1 The statement made here is relatively straight-forward but not wrong. You may take it out if you are pressured for space.*

***We prefer to leave this section in. We agree it is straightforward but everyone still seems to be doing the log transformation and we want to discourage its use for UMI data.***

*- 5: 2.6 Generalized PCA for dimension reduction of sparse counts, 2.7 Deviance residuals provide fast approximation to GLM- PCA, 2.10 Computational efficiency of multinomial models*

*-- 5.1 The points made here is nice, again it crucially depends on the negative control analysis that I suggest in comment 3.1.*

***We address this point under Comment 3.1 above.***

*-- 5.2 Scalability and run time are clearly important issues for PCA as PCA is often used as a reduction before kNN-graph based methods or other dimension reductions are used. Please add 2.10 closer or into 2.6, I was confused by not finding it when reading this section.*

***We moved the computational speed to Section 2.8 (right after the discussion of residuals) and now include a sentence referring to it in Section 2.6.***

*-- 5.3 Approximation of GLM-PCA by PCA on deviance residuals: The concept is clear here, but clearly the remainder of the optimisation is potentially biased as the the wrong noise model is assumed in the vanilla PCA implementation that is used after the feature space is transformed to residuals. It would be important to discuss accuracy with respect to PCA and GLM-PCA of this residual-based PCA, for example in simulation studies.*

***We include PCA of residuals in all benchmarking. Results are shown in Figures 5a,S3,S7a,S8,S9 and Tables S1 and S2.***

*- 6: 2.8 Feature selection using deviance*

*- The intuition why this would work is clear. However, I find that the motivation of how it is working better is lacking, if you take expression strength as a gold standard, why not use expression strength in the first place in practice?*

***This is now results Section 2.9. We do not consider expression strength as a gold standard. Our perspective is that deviance provides a statistically rigorous approach to feature selection based on the appropriate multinomial model. Whether the genes chosen overlap or don’t overlap with existing ad-hoc methods like highly variable genes or high expression doesn’t affect the validity of the idea. To further support our argument, we have made additional comparisons of the gene filtering methods, detailed in Results and discussion Section 2.9. In particular, we now show that deviance does not merely identify highly expressed genes in both a simulation (Figure S5) and a real dataset (Muraro et al., Figure S6). The reason high deviance genes overlapped so much with highly expressed genes in the Zheng 8eq dataset is because the highly expressed genes have distributions that deviate from a model of constant expression across cells, as illustrated in the below ridge plot. These genes were chosen to be in the top 100 highly expressed, top 150 high deviance, but >2,000 ranking according to HVG.***

*- 7: 2.9 Multinomial models improve unsupervised clustering*

***This is now the Results and discussion Section 2.10***

*-	7.1 The argument presented here is based on k-means in the latent space. I would like to really emphasize that k-means in the latent space is not common practice for scRNA-seq, almost every large study presents clusterings from the louvain/leiden method. Please benchmark on this, otherwise you may risk to appear to cherry-pick the benchmarking scenario.*

***Unless otherwise noted, all clustering results were with Seurat which uses a Louvain algorithm.***

*-	7.2 You again make the point that feature selection on deviance performs similarly to total expression.*

***The updates to Results and discussion Section 2.9 (based on Reviewer #2 comment 6) now describe and explain that it is not a problem that deviance and highly expressed identify similar sets of genes on these data.***

*Minor comments:*

*- "(often > 90% of the data, sometimes called dropouts)." I would avoid the use of drop-out unless you discuss that this is not drop-out in the zero-inflated sense right at this point. The fact that dropout is used for the occurrence of a zero in a simple sampling process (such as multinomial as suggested here) is a major confusion in the field and we can only rid ourselves from this confusion by coherently avoiding or redefining it at every occurrence.*

***We have now removed all mentions of dropout except in the context of “highly dropout genes”, which is a feature selection method we briefly discuss.***

*- Could you state the number of PCR cycles for library amplification used in the most common protocols? This would be helpful to guide intuition as to how large the effects can be here.*

***We now include the sentence: “For example, the popular 10x genomics protocol uses 14 cycles [cite Zheng]”***

*- "The statistical approaches described here have not been validated against scRNA-Seq data without UMIs, such as SMART-Seq2 and other plate protocols [8], since non-UMI data contain PCR duplicates. To apply the ideas to these data, one would need to be able to infer the UMI counts for data with PCR replicates [7]." I do not see the point of this section and would remove it. Clearly, all your statements are about UMI data.*

***We have now removed this from the manuscript.***

**Second round of review**

**Reviewer 1**

The authors have done a very good job in addressing all of my previous concern, except for concern 4.). Regarding this concern I would like to specify that I am merely interested in the genes (with high loadings) underlying the separation of the data along the principle components. They argue that conventional PCA ends up identifying sequencing-depth related variability as the major principle component. I would like to see evidence for this, since my expectation is that conventional PCA will separate the data based on biological variability (related to the expression of specific genes) along the major principle components. Hence, I would like to see exactly what the authors outline in their response. If the genes with the top loadings of the major principle components are markers of specific cell types, or, more generally, markers of biological variability this argues against the claim that conventional PCA separates the data based on sequencing depth along the major principle component, and the authors cannot make this claim.

**Reviewer**

Summary: Overall, the authors took big steps in the right direction. The manuscript would however benefit from further validation that I already suggested before and clarified here again. I raised those concerns below. As stated in the initial summary, the selling point of this manuscript is a vigorous analysis of statistics here, there are many studies, blog posts and talk with weak evidence on similar questions. I would like to encourage the authors to take this opportunity to take the last steps to make this manuscript really count towards shedding light on these important issues.

>>Review of Feature Selection and Dimension Reduction for Single Cell RNA-Seq based on a Multinomial Model

>>Major comments:

>>- 1: 2.2 UMI count distribution differs from reads

The authors make the point that UMI data is not zero-inflated. This notion is nowadays

widely believed to be true in the field but has not been well supported by published

studies so that there is still discussions about this. Exploring this issue on UMI data that

has not been deduplicated is a very good idea. I would ask the authors to really put

more effort into quantifying this effect though. This statement is about choice of noise

model which has vast effects on differential expression analysis and p-values, any

hypothesis presented in this context should be backed by more solid analysis. Right

now, the authors present histograms of a single gene, this is clearly not sufficient. I do

agree with the authors that ZI is likely a PCR-artefact, but finding evidence for this has

been an issue, which is why other studies has stayed clear of touching this issue. This

is an important point to make and a good contribution of the manuscript so I would really

encourage the authors to work on this. Some ideas, but please do not let this constrain

your creativity: model selection based on likelihood-based model fits, moment-based

statistics (e.g. number of zeros versus mean).

>We now provide Figure S2 (and discussion in Results and discussion Section 2.3)

where we compare goodness-of-fit of 7 different possible distributions (including

some zero inflated) to both UMI counts and read counts of the same data. Here,

we demonstrate that the multinomial, Dirichlet-multinomial, and Poisson (as approximation to multinomial) distributions fit best. When applied to read counts, the zero-inflated lognormal was the best fitting distribution followed by the Dirichlet-multinomial.

I do not think that null model fits are appropriate to compare noise models as you ignore the variance originating in the biological structure of the data set: cell types etc. This likely results in your distribution choice to be biased to distributions that can handle strong unaccounted confounding. I think that the comparison in Fig. S2 should be performed with full models accounting for all known confounders.

>>- 2: 2.3 Multinomial sampling distribution for UMI counts

>>-- 2.1 You state that a multinomial distribution can be approximated by independent

poisson and negative binomial distribtions. This is an important point as negative

binomial noise is a common choice for modelling RNA-seq and scRNA-seq data. As this

is a distribution centric paper, it would be nice to supply some details on how well this

approximations work, ie at what number of samples they break down etc.

>We discuss this in Methods 4.1, the paragraph beginning “First, note that if

correlation is ignored...”. The approximation breaks down when the total counts

of a cell are very small, when a single feature (gene) has a probability much larger

than all the others, or when the number of features is small. We cite references

from the statistical literature that rigorously quantify and put bounds on the

approximation error. In addition, we now provide a more intuitive explanation in

the manuscript.

Ok.

>>-- 2.1 You base the main point of this section on the argument that the multinomial fits

two characteristics of the data:

>>* fraction of zeros is inversely related to total number of UMIs

>>* the probability of zero counts is a decreasing function of the mean

>>--- 2.1.1 Both arguments are related to first moments of the distribution per gene or

across the sample. I am not sure how well they would serve to distinguish noise models

as the major distinction between unimodal noise models lies in the second and higher

order moments.

>We now provide Figure S2 (and Results and discussion Section 2.3) to address

this point. We have now expanded our analysis to assessing goodness-of-fit

(using Bayesian information criterion or BIC) for 7 sampling distributions to each

of the datasets containing biological replicate negative controls.

See my above comment on S2, I do not think that GLM fits based on a null model for the location parameter are sufficient.

>>--- 2.1.2 More importantly, you do not compare these distributions against other noise

models. This is a weakness of this analysis. It is hard to argue that one should use

multinomial noise models or their approximations because they fit to some more or less

arbitrarily selected summary statistics. I would suggest to compare to other noise

models (normal, log-normal, hurdle etc.). Again likelihood-based model selection may

be an option to select between models.

>We now provide Figure S2 (and Results and discussion Section 2.3) to address

this point.

See pervious comment.

>>- 3: 2.4 Normalisation and log-transformation distorts UMI data

>>-- 3.1 You argue that artefacts occur in dimension reduction by showing that the first

PC of log transformed data is correlated with fraction of zeros. You however fail to show

whether this also occurs in the non-log-transformed control of your analysis that you

should have done. Can you please add this control analysis?

>We now report Pearson correlation values for technical and biological

(monocytes) replicates between PC1 and zero fraction both before and after log

transformation of CPM values (Results and discussion Section 2.4 in the last

paragraph). Note, log transforming reduces the correlation between PC1 and zero

fraction. After centering and scaling, this is roughly equivalent to PCA on

approximate multinomial Pearson residuals which we regard as an approximation

to GLM-PCA.

Ok.

>>-- 3.2 The major reason for log-transformation is a feature engineering perspective on

the data: It reduces the difference in variance contributed by each gene that is related to

its mean (mean-variance relation ship of count distributions) and thereby reduces the

bias of embeddings and clusterings to depend mostly on highly expressed genes. It

would be important to discuss this.

>We had previously discussed the motivation for log transforming in the Methods Section 4.2 in that it facilitates interpreting differential expression in terms of fold changes. We now include the following in that section: “Also, for count data, the variance of each gene is a function of its mean, and log-transformation can help to prevent highly expressed outlier genes from overwhelming downstream analyses.”

You could add the cumulative distribution of genes across variance explained: x axis are genes ordered by variance contributed and y axis is total variance explained by all genes up to that x coordinate. You could compare this between PCA, log-transform followed by PCA and by GLM-PCA so allow the user to judge the number of genes the effectively contribute to the embedding in your method and the known reference methods.

>>- 4: 2.5 Zero inflation is an artifact of log-normalization

>>-- 4.1 The statement made here is relatively straight-forward but not wrong. You may

take it out if you are pressured for space.

>We prefer to leave this section in. We agree it is straightforward but everyone still seems to be doing the log transformation and we want to discourage its use for UMI data.

Ok.

>>- 5: 2.6 Generalized PCA for dimension reduction of sparse counts, 2.7 Deviance

residuals provide fast approximation to GLM- PCA, 2.10 Computational efficiency of multinomial models

>>-- 5.1 The points made here is nice, again it crucially depends on the negative control analysis that I suggest in comment 3.1.

>We address this point under Comment 3.1 above.

Ok.

>>-- 5.2 Scalability and run time are clearly important issues for PCA as PCA is often

used as a reduction before kNN-graph based methods or other dimension reductions

are used. Please add 2.10 closer or into 2.6, I was confused by not finding it when

reading this section.

>We moved the computational speed to Section 2.8 (right after the discussion of

residuals) and now include a sentence referring to it in Section 2.6.

Ok.

>>-- 5.3 Approximation of GLM-PCA by PCA on deviance residuals: The concept is clear

here, but clearly the remainder of the optimisation is potentially biased as the the wrong

noise model is assumed in the vanilla PCA implementation that is used after the feature

space is transformed to residuals. It would be important to discuss accuracy with

respect to PCA and GLM-PCA of this residual-based PCA, for example in simulation

studies.

>We include PCA of residuals in all benchmarking. Results are shown in Figures 5a,S3,S7a,S8,S9 and Tables S1 and S2.

The results presented to not show accuracy of this proposed approximation in simulations. Please show this and show how / where this approximation breaks.

>>- 6: 2.8 Feature selection using deviance

>>- The intuition why this would work is clear. However, I find that the motivation of how it

is working better is lacking, if you take expression strength as a gold standard, why not

use expression strength in the first place in practice?

>This is now results Section 2.9. We do not consider expression strength as a gold standard. Our perspective is that deviance provides a statistically rigorous approach to feature selection based on the appropriate multinomial model. Whether the genes chosen overlap or don’t overlap with existing ad-hoc methods like highly variable genes or high expression doesn’t affect the validity of the idea.

It does not affect the validity, but I hope you strive for a useful rather than merely valid idea. Tt is valid to ask for empirical validation of this idea as you cannot prove that you statistical model is correct. The entire field of differential expression analysis revolves around statistical motivation and empirical validation. Focus of this validation on highly expressed genes was not satisfactory in the previous version.

>To further support our argument, we have made additional comparisons of the gene filtering methods, detailed in Results and discussion Section 2.9. In particular, we now show that deviance does not merely identify highly expressed genes in both a simulation (Figure S5) and a real dataset (Muraro et al., Figure S6). The reason high deviance genes overlapped so much with highly expressed genes in the Zheng 8eq dataset is because the highly expressed genes have distributions that deviate from a model of constant expression across cells, as illustrated in the below ridge plot. These genes were chosen to be in the top 100 highly expressed, top 150 high deviance, but >2,000 ranking according to HVG.

The five genes that you show seem zero-inflated – would you agree? My understanding of your motivation to use the deviance as a feature selection criteria is that it captures strong confounding effects that increase the variance of the data, e.g. that make the distribution bi-modal if there are two latent groups present or that the variance is simply inflated in a weaker case. This plot does not seem to suggest that this is the case here (unless one group is entirely zero in each of those genes).

A reasonable step towards validation would be comparison to previous methods, ie. look at confusion tables between the features you select and other selection methods.

>>- 7: 2.9 Multinomial models improve unsupervised clustering

>This is now the Results and discussion Section 2.10

>>- 7.1 The argument presented here is based on k-means in the latent space. I would

like to really emphasize that k-means in the latent space is not common practice for

scRNA-seq, almost every large study presents clusterings from the louvain/leiden

method. Please benchmark on this, otherwise you may risk to appear to cherry-pick the

benchmarking scenario.

>Unless otherwise noted, all clustering results were with Seurat which uses a Louvain algorithm.

Ok.

>>- 7.2 You again make the point that feature selection on deviance performs similarly to

total expression.

>The updates to Results and discussion Section 2.9 (based on Reviewer #2 comment 6) now describe and explain that it is not a problem that deviance and highly expressed identify similar sets of genes on these data.

See 6.

>>Minor comments:

>>- "(often > 90% of the data, sometimes called dropouts)." I would avoid the use of drop-

out unless you discuss that this is not drop-out in the zero-inflated sense right at this

point. The fact that dropout is used for the occurrence of a zero in a simple sampling

process (such as multinomial as suggested here) is a major confusion in the field and

gene we can only rid ourselves from this confusion by coherently avoiding or redefining it at every occurrence.

>We have now removed all mentions of dropout except in the context of “highly dropout genes”, which is a feature selection method we briefly discuss.

I discourage any word invention around the word dropout.

>>- Could you state the number of PCR cycles for library amplification used in the most common protocols? This would be helpful to guide intuition as to how large the effects can be here.

>We now include the sentence: “For example, the popular 10x genomics protocol

uses 14 cycles [cite Zheng]”

Ok.

>>- "The statistical approaches described here have not been validated against scRNA-

Seq data without UMIs, such as SMART-Seq2 and other plate protocols [8], since non-

UMI data contain PCR duplicates. To apply the ideas to these data, one would need to

be able to infer the UMI counts for data with PCR replicates [7]." I do not see the point

of this section and would remove it. Clearly, all your statements are about UMI data.

>We have now removed this from the manuscript.

Ok.

**Authors Response**

**Point-by-point responses to the reviewers’ comments:**

*Reviewer #1: The authors have done a very good job in addressing all of my previous concern, except for concern 4.). Regarding this concern I would like to specify that I am merely interested in the genes (with high loadings) underlying the separation of the data along the principle components. They argue that conventional PCA ends up identifying sequencing-depth related variability as the major principle component. I would like to see evidence for this, since my expectation is that conventional PCA will separate the data based on biological variability (related to the expression of specific genes) along the major principle components. Hence, I would like to see exactly what the authors outline in their response. If the genes with the top loadings of the major principle components are markers of specific cell types, or, more generally, markers of biological variability this argues against the claim that conventional PCA separates the data based on sequencing depth along the major principle component, and the authors cannot make this claim.*

***We now explain in last paragraph of section 2.4 that we are not claiming that conventional PCA always ends up identifying sequencing-depth related variability as the dominant PC and that instead we are demonstrating, both theoretically and empirically, how differences in sequencing-depth can lead to unwanted variability after the log2(1+x) transformation. We also examined the loadings for the first PC, as suggested. Specifically we ran PCA on log2(1+CPM) of the same random 5,000 genes as in Figure 3b (the monocytes biological replicates dataset). The figure below shows the loadings for all the genes with marker genes for the monocyte subpopulations highlighted. We note that it does not appear that this group of genes is driving the first PC. We also note that there is no clear cluster of genes that might be driving the variability consistent with a global effect such as sequencing-depth.***

| *gene* | *hgnc_symbol* | *loading on PC1* |
| --- | --- | --- |
| *ENSG00000087086* | *FTL* | *-0.07333616* |
| *ENSG00000241106* | *HLA-DOB* | *0.06607392* |
| *ENSG00000180817* | *PPA1* | *0.06530296* |
| *ENSG00000057608* | *GDI2* | *0.06353922* |
| *ENSG00000205581* | *HMGN1* | *0.06040456* |
| *ENSG00000196154* | *S100A4* | *-0.05950092* |
| *ENSG00000115091* | *ACTR3* | *0.05881087* |
| *ENSG00000086598* | *TMED2* | *0.05824197* |
| *ENSG00000118292* | *C1orf54* | *0.05589368* |
| *ENSG00000139626* | *ITGB7* | *0.05527963* |
| *ENSG00000155367* | *PPM1J* | *0.05445288* |
| *ENSG00000181163* | *NPM1* | *0.05409598* |
| *ENSG00000140968* | *IRF8* | *0.05339455* |
| *ENSG00000152234* | *ATP5F1A* | *0.05237757* |
| *ENSG00000165678* | *GHITM* | *0.05224784* |
| *ENSG00000100243* | *CYB5R3* | *0.05215911* |
| *ENSG00000156976* | *EIF4A2* | *0.05205408* |
| *ENSG00000213585* | *VDAC1* | *0.05194996* |
| *ENSG00000075415* | *SLC25A3* | *0.05150328* |
| *ENSG00000196405* | *EVL* | *0.05115054* |

*Reviewer #2:*

*Major comments:*

*I do not think that null model fits are appropriate to compare noise models as you ignore the variance originating in the biological structure of the data set: cell types etc. This likely results in your distribution choice to be biased to distributions that can handle strong unaccounted confounding. I think that the comparison in Fig. S2 should be performed with full models accounting for all known confounders.*

***We re-ran the model fitting on the same datasets this time including the following covariates in addition to total count: fraction of zeros, percent mitochondrial RNA, and cell cycle phase scores for S and G2/M using gene lists provided by Seurat. We fit Poisson, negative binomial, zero inflated Poisson, and zero inflated lognormal models. We considered Poisson an approximation to multinomial, and negative binomial an approximation to Dirichlet-multinomial. As shown in the below figures, the ordering of models based on BIC is unchanged whether or not covariates are included. Moreover, in the UMI data, adding covariates actually made the BIC score worse (because the increase in number of parameters associated with covariate adjustment did not substantially improve the goodness-of-fit to the data).***

***Because both of these datasets (Tung and Zheng monocytes) are negative controls- the cells were purified so that there is only one cell type and all cells were processed in a single batch- we do not expect to see biologically interesting variability. As a result, the figures included here show very similar results to those in Figure S2 and we did not include them in the manuscript. However, if the reviewers disagree we are happy to add these figures.***

*Figure 1 - Tung UMI counts*

*Figure 2 - Tung read counts*

*Figure 3 - Zheng monocytes UMI counts*

*Figure 4 - Zheng monocytes read counts (nb had numerical convergence problems)*

*-- 3.2 You could add the cumulative distribution of genes across variance explained: x axis are genes ordered by variance contributed and y axis is total variance explained by all genes up to that x coordinate. You could compare this between PCA, log-transform followed by PCA and by GLM-PCA so allow the user to judge the number of genes the effectively contribute to the embedding in your method and the known reference methods.*

***We need clarification on this request. Suppose we apply PCA to a matrix Y with genes in rows and cells in columns and obtain the factorization Y = VU’ with U a matrix with principal components in the columns and V the loadings in the (orthonormal) columns. In terms of column or rows of Y, V and U, what is the plot requested?***

*-- 5.3 The results presented do not show accuracy of this proposed approximation in simulations. Please show this and show how / where this approximation breaks.*

***We now describe in results section 2.7 and methods section 4.4 that we simulated 150 cells in three clusters of 50 cells each with 5,000 genes, of which 500 were differentially expressed across clusters (informative genes). We also created two batches, batch 1 (circles) with total counts=1,000 and batch 2 (triangles) with total counts=2,000. Each cluster had an equal number of cells in the two batches. We then ran GLM-PCA on the raw counts, PCA on log2(1+CPM), PCA on deviance residuals (“pca_dr”), and PCA on Pearson residuals (“pca_pr”) with number of dimensions=2. As shown in the below plots (now included as figure S11 in manuscript), the first PC aligned with the batch effect in standard PCA, while the second PC only separated one of the clusters. GLM-PCA correctly recovered the three clusters with the batch effect removed. The two residuals-based approximations to GLM-PCA also recovered the correct three clusters. However, the clusters were not as tightly grouped. One of the batches (circles) tended to be closer to the center of the plot whereas within each cluster, the batch 2 (triangles) tended to be further away. This shows that if the batch effect is extreme, it could lead the analyst to conclude there are as many as 6 clusters whereas in reality there are only 3 when using the residuals-based approximation. However, note that the clustering quality is still much higher than traditional PCA, and the speed is 10x faster than GLM-PCA.***

*- 6: 2.8 Feature selection using deviance*

*The five genes that you show seem zero-inflated – would you agree? My understanding of your motivation to use the deviance as a feature selection criteria is that it captures strong confounding effects that increase the variance of the data, e.g. that make the distribution bi-modal if there are two latent groups present or that the variance is simply inflated in a weaker case. This plot does not seem to suggest that this is the case here (unless one group is entirely zero in each of those genes).*

*A reasonable step towards validation would be comparison to previous methods, ie. look at confusion tables between the features you select and other selection methods.*

***We now include confusion tables comparing highly variable genes, highly expressed genes, and high deviance genes for the top 1,000 genes in table S3. We also point out that none of the top 1,000 highly expressed genes are flagged as highly variable or high deviance. Among the 1,000 top deviance genes 918 are also found to be highly variable so these two criteria agree on this simulation.***

***We also include confusion tables for the Muraro dataset (table S4) and note that of the top 1,000 genes that are highly expressed, 167 are also high deviance and highly variable. A further 200 are high deviance but not highly variable. In total, 633 of the high deviance genes are not in the top 1,000 highly expressed genes. The overlap between high deviance and highly variable genes is also limited for this dataset: 439 of the high deviance genes are not highly variable and vice- versa. Of these, 239 high deviance genes are neither highly expressed nor highly variable.***

**Third round of review**

**Reviewer 1**

The authors have satisfactorily addressed my last remaining concern.

**Reviewer 2**

The authors have addressed my concerns very well, thank you. My minor question on cumulative gene distributions IMHO does not warrant another review round and I am happy with the state of the manuscript.
